# Concomitant body-wide trauma patterns in patients with head and neck injuries: a comparison based on the trauma register DGU® by the German trauma society and the dortmund maxillofacial trauma registry

**DOI:** 10.1186/s40001-025-02636-x

**Published:** 2025-05-08

**Authors:** Ákos Bicsák, Fatma Topcuoglu, Stefan Hassfeld, Rolf Lefering, Lars Bonitz, Jens-Peter Stahl

**Affiliations:** 1 Clinic for Oral- and Maxillofacial Surgery, Regional Plastic Surgery, Dortmund General Hospital, Chair of the University of Witten-Herdecke, Muensterstrasse 240, 44145 Dortmund, Germany; 2 Health Faculty, University of Witten/Herdecke, Alfred-Herrhausen-Strasse 45, 58453 Witten, Germany; 3 Clinic for Traumatology, Hand and Reconstructive Surgery, Dortmund General Hospital, Muensterstrasse 240, 44145 Dortmund, Germany; 4 Institute for Research in Operative Medicine (IFOM), University Witten/Herdecke, Ostmerheimer Strasse 200, Haus 38, D-51109 Cologne, Germany; 5 Trauma Register GDU® of the German Trauma Society (DGU), Lead By AUC - Akademie der Unfallchirurgie GmbH, Emil-Riedel-Strasse 5, 80538 Munich, Germany

**Keywords:** Facial bone fractures, Torso injuries, Spine injury, Thoracic injury, Pelvic injury

## Abstract

**Introduction:**

Interdisciplinary trauma patients with multiple injuries require special attention from the first minute of care. This study aimed to determine the overall injury distribution in patients with head and neck injury (HNI) with respect to the head and neck injury location.

**Methods:**

Data on patients with HNI were collected in the Dortmund Maxillofacial Trauma Register 2007–2017 based on a review of patient radiographs and files. All patients with concomitant injuries to central body parts were selected and analysed further by an expert traumatologist collecting data on GCS, ISS, injuries and outcome. Further comparisons with data from the German TraumaRegister DGU® 2007–2022 were acquired and used to determine whether the patient group with head and neck injuries differed from other injured patients.

**Results:**

A total of 70.212 patients with head and neck injuries were identified from the 344.754 reported cases in the TraumaRegister DGU®. Among the 344,754 patients, 6127 were registered with mandibular injuries (1.8% of the total population/8.7% of patients with HNI), panfacial injuries were reported in 10,001 patients (2.9%/14.2%), and midfacial injuries were reported in 42,045 patients (12.2%/59.9%). Injuries to the thorax (39.5% of non-HNI patients vs. 35.4% of HNI patients), abdomen (10.8% vs. 6.7% of HNI patients), and extremities (25.6% non-HNI vs. 20.1% HNI, respectively), spine (29.8% non-HNI patients vs. 27.0% HNI patients), lower extremities (26.1% vs. 22.3%, respectively), and iliac bone (16.5% non-HNI patients vs. 13.6% HNI patients) and upper extremities (29.0% non-HNI patients and 33.8% HNI patients). A total of 289 severely injured patients from 7010 head and neck injuries were selected from the Dortmund Maxillofacial Trauma Register. The mechanism of the injuries included falls (*n* = 143; 49.5%), road traffic accidents (75, 26.0%), interpersonal violence (48, 16.6%), work-related accidents (14, 4.8%), sports accidents (7, 2.4%) and other causes (2, 0.7%). No in-hospital deaths were reported in this population. Seven of the 289 patients (2.4%) needed reanimation upon arrival at the emergency room. In this group, the average ISS = 50.3 (Injury Severity Score), and the GCS = 7.1 (Glasgow Coma Scale). Six of the 7 patients had a calvarial fracture (86%), and injuries to the cervical, thoracic and lumbar spine were detected in 1, 2 and 2 patients (14%, 28% and 28%, respectively). Ventilation was applied in 4 patients (57%). Overall, most soft tissue injuries were identified in the face (128) and head areas (57), followed by the thorax and back areas (54 and 28). The most joint injuries were found in the hip, shoulder and radiocarpal and hand regions (11, 10 and 10 injuries, respectively). Most cases were considered severe. The body-wide fracture distribution was similar: most fractures occurred in the calvaria (227) or thoracic area (27 multiple rib fractures and 15 single rib fractures). Similarly, the rates of fractures in the thoracic spine (15), lumbar spine (11) and pelvic bone (9) were high. The 289 analysed patients accounted for 4.1% of all patients with head and neck injuries. Together with the 2.47% of patients with CSI (cervical spine injury), all HNI patients with any central body injury accounted for nearly 6.5% of all HNI patients. The results are presented in heatmaps.

**Conclusions:**

Patients with HNI tend to have slightly different injury pattern in the whole body than non-HNI patients. Head and neck injuries in different locations show different patterns of concomitant injuries. Panfacial fractures, forehead and lateral and central midfacial injuries combine with spine injuries, central midface, mandibular and dentoalveolar injuries were not observed with hip fracture. Injuries to the thorax were not diagnosed in case of dentoalveolar injuries.

**Supplementary Information:**

The online version contains supplementary material available at 10.1186/s40001-025-02636-x.

## Introduction

The initial trauma assessment, both at the trauma site and in the emergency room, determines the further fate of the patients. Missing or false initial diagnosis of injuries can have severe consequences [1–3]. Detecting and identifying injury patterns and combinations can facilitate quicker recognition process that ensures that patients are managed adequately and in a timely manner [4, 5].

Our Hospital has a highest level Interdisciplinary Trauma Centre. As regulated, it cooperates with the Trauma Register DGU® of the German Trauma Society (Deutsche Gesellschaft für Unfallchirurgie, DGU) [6]. This organization collects data from severely injured patients per the criteria of the Abbreviated Injury Scale (AIS) [7]. Input is received from approximately 700 participating centres located in Germany, Austria, Switzerland, and a few centres in other countries. The data set includes injury data, vital parameters, preclinical and clinical data, and diagnostic, therapeutic and outcome data. Data access and acquisition are possible for participating centres for research purposes.

The Department of Maxillofacial Surgery has its own trauma databank (Dortmund Maxillofacial Trauma Registry) that contains data based on the current AO classification of facial fractures [8–14].

A pattern can be recognized in the localization of multiple injuries found exclusively in the head and neck region, as well as head and neck injuries associated with cervical spine injuries [15–19]. Based on these results, this study aims to evaluate the patient population admitted to our hospital for head and neck injuries between 1.1.2007 and 31.12.2017 to determine whether similar injury patterns exist, such as in patients with cervical spine injuries [20]. Our hypothesis is that we can find a correlation between bodywide injury patterns and different facial fracture distribution patterns. Secondary goal is to compare the results of the Dortmund Maxillofacial Trauma Registry with the data retrieved from the Trauma Register DGU® (for example, definition of reanimation, polytrauma and fracture classification).

## Materials and methods

### Ethics commission, patient consent, DGU review

The Ethics Committee of the University of Witten/Herdecke approved this study (172/2017). The DGU has found the study as reasonable and approved the data acquisition. The study was conducted in accordance with the Declaration of Helsinki, Good Clinical Practice, and the laws and regulations of the European Union, the German Federal Republic, the state Northrhine–Westfalia, the University of Witten/Herdecke, Dortmund General Hospital, and the German Trauma Register DGU®.

### Treatment procedure

**Fig. 1:**
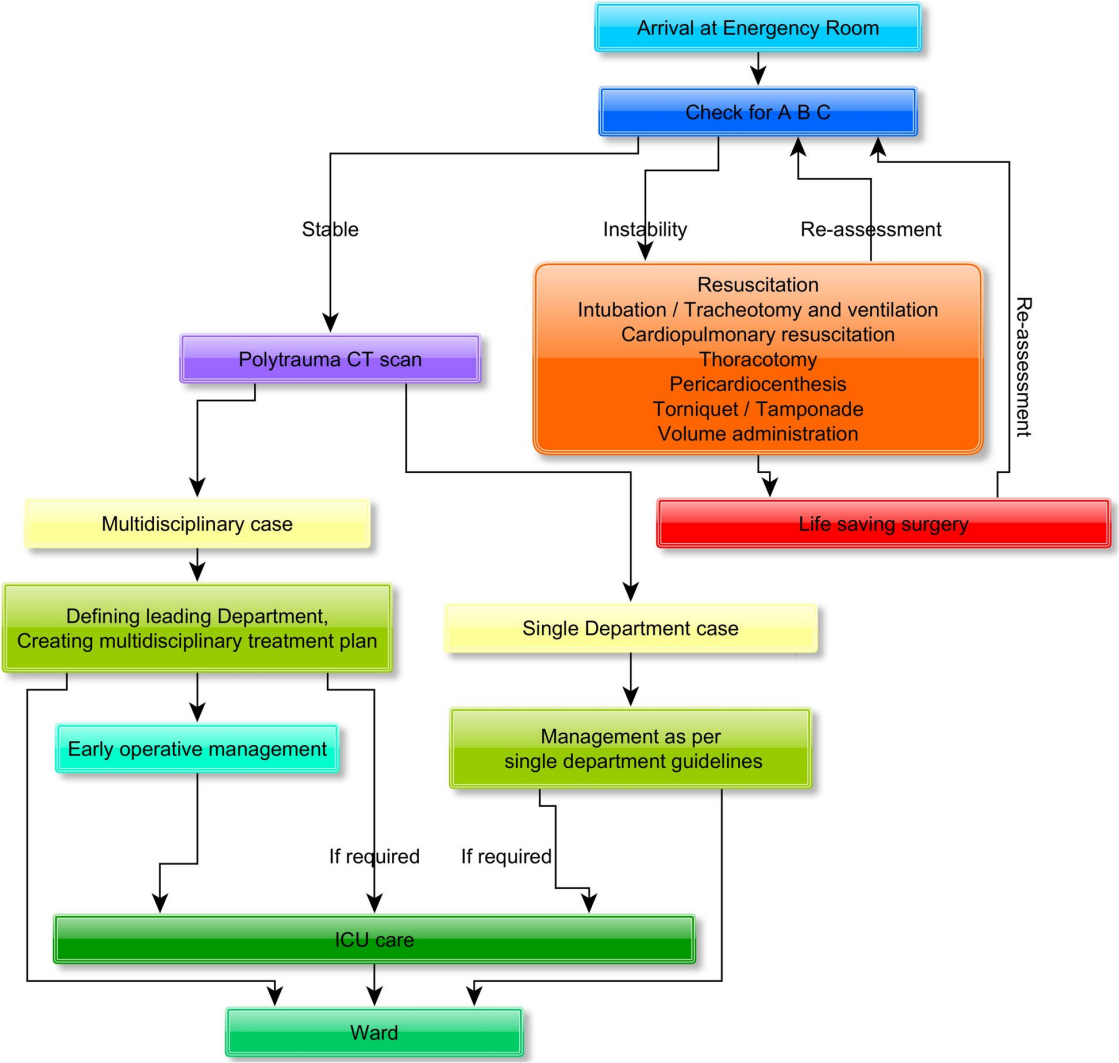
Emergency room flow chart (based on the actual German guidelines [21, 27]). After arrival at the ER, the patient is checked for airways, breathing, and circulation. If stabilization is necessary, the patient is stabilized (by a team of intensive care specialists, traumatologists, and other specialists, as needed). A polytrauma CT scan is performed as soon as possible to allow the patient to recover from all injuries. After initial diagnostics are completed, further treatment needs are defined and planned

The treatment procedure is based on the current German emergency room guidelines [21] and is presented in Fig. 1. In accordance with the current emergency room guidelines, patients are initially assessed by a well-trained traumatologist assisted by clinicians in other disciplines, such as radiology, maxillofacial surgery, neurosurgery, and vascular surgery. The AO classification systems for bone fractures and craniomaxillofacial fractures, the Glasgow Coma Scale (GCS), and the injury severity score (ISS) are widely used to standardize the assessment, as they provide excellent clinical assessment and make clinical research comparable [4, 15, 22]. After immediate measures, an individual, interdisciplinary treatment plan is agreed. Each participating discipline plans its further treatment based on own guidelines.

### Data collection and patient pool

Two data sources were used for this study.

#### Dortmund maxillofacial trauma registry

The Dortmund General Hospital hosts this in-centre registry based on REDCap (REDCap Consortium, Nashville, Tennessee, USA), and serves as an electronic data capture system.

The fracture classification is based on the AO classification systems [8–14] provided by the AO Foundation (Gesellschaft für Allgemeine Osteosynthesefragen, Davos, Switzerland). This classification system matches the needs for therapeutic decisions required by oral and maxillofacial surgeons and has more levels of detail levels than the AIS score system (see below). Patient groups were created to cover major head and neck injury distribution patterns (which also allowed matching with the AIS score).

The registry data were filtered for patients with concomitant injuries (polytrauma or non-polytrauma patients were included). This group of patients was further analysed by trained trauma surgeon, who listed soft tissue, joint, and bony injuries in all body regions. The injury distribution was stratified statistically and visually presented for all patient groups.

For easier reporting we summarized thoracal, abdominal, spinal and hip injuries as “central injuries”.

#### TraumaRegister DGU®

The following description is adopted from the guidance document of the TraumaRegister DGU® [23]:

The TraumaRegister DGU® of the German Trauma Society (Deutsche Gesellschaft für Unfallchirurgie, DGU) was founded in 1993. The aim of this multicentre database is pseudonymized and standardized documentation of severely injured patients. Data are collected prospectively in four consecutive time phases from the accident site until discharge from the hospital: (A) prehospital phase; (B) the emergency room and initial surgery; (C) the intensive care unit; and (D) discharge. The documentation includes detailed information on demographics, injury patterns, comorbidities, pre- and in-hospital management, the course in the intensive care unit, and relevant laboratory findings, including data on transfusions and the outcome of each individual. The inclusion criterion is admission to the hospital via the emergency room with subsequent ICU/ICM care or reaching the hospital with vital signs and dying before admission to the ICU.

The infrastructure for documentation, data management, and data analysis is provided by the AUC—Academy for Trauma Surgery (AUC—Akademie der Unfallchirurgie GmbH), a company affiliated with the German Trauma Society. The scientific leadership is provided by the Committee on Emergency Medicine, Intensive Care and Trauma Management (Section NIS) of the German Trauma Society. The participating hospitals submit pseudonymized data into a central database via a web-based application. Scientific data analysis is approved according to a peer review procedure laid down in the publication guidelines of the TraumaRegister DGU®. The participating hospitals are primarily located in Germany (90%), but an increasing number of hospitals from other countries contribute data as well (at the moment, from Austria, Belgium, China, Finland, Luxembourg, Slovenia, Switzerland, The Netherlands, and the United Arab Emirates). Over 38,000 cases from almost 700 hospitals are entered into the database annually. Participation in the TraumaRegister DGU® is voluntary. For hospitals associated with Trauma Netzwerk DGU®, however, the entry of at least a basic data set is obligatory for quality assurance reasons [23].

For the purposes of this study, data from Germany, Switzerland, and Austria were selected, as these countries have very similar developmental and cultural backgrounds to our centre. Patients documented from 2007 to 2022 were considered. Patients who transferred out early (< 48 h) were excluded, because they appeared as transfer-in cases at another hospital. The data were then compared with data from our centre.

The definitions and terms used by the Trauma Register DGU® and Dortmund Maxillofacial Trauma Registry were checked for consistency. If there was a difference in the definitions, the local data analysis was adapted to match the DGU-term.

### Statistics

The statistical equations were performed with SPSS (version 29, IBM Inc., Armonk, NY, USA). The descriptive analysis was performed with the numbers of cases and percentages for categorical data and the means with standard deviations (SDs) for metric data. The chi-square test was used for group comparisons, and an independent *t* test was used to analyse continuous numerical data. The level of significance was considered p < 0.05.

## Results

### Results from the TraumaRegister DGU®

The TraumaRegister DGU® was analysed from 2007 to 2022. In the TraumaRegister DGU, a total of 344,754 patients met the inclusion criteria. Ten percent of these patients were reportedly from Austria and Switzerland, and 90% were from German trauma centres. Among them, 70,212 (20.4%) presented with head and neck injury (HNI). The yearly rate of HNI varied between 19.0% and 20.5% in more extended series; therefore, we assumed that it was approximately stable during the observation period. The incidence was 7/100,000 p.a. This incidence was calculated for Germany and is based on the years 2013–2018, where the registry had nearly complete coverage in Germany. Among the 344,754 patients, 6,127 were registered with mandibular injuries (1.8% of the total population/8.7% of patients with HNI), panfacial injuries were reported in 10,001 patients (2.9%/14.2%), and midfacial injuries were reported in 42,045 patients (12.2%/59.9%). The mean age of all injured patients was 52.2 ± 22.8 years, and that of patients with HNI was 50.4 ± 20.7 years (p < 0.05).

A total of 75.9% of the HNI patients presented in level 1 trauma centres compared with 62.5% of non-HNI patients (p < 0.05). The HNI group had a slightly higher percentage of children (< 16 years of age) (3.7% vs. 3.4% of all patients) and adults (16–59 years) (59.9% vs. 56.8% of all patients), but a slightly lower percentage of elderly patients (13.4% non-HNI vs. 12.1% HNI (70–79 years) and 13.4% non-HNI vs. 11.9% HNI (80 + years)) (p < 0.05).

The aetiology of HNI also differed slightly from that of non-HNI injuries: less suicide (4.0% vs. 4.5% non-HNI), fewer motorcycle accidents [8.9% vs. 13.6% non-HNI, (*p* < 0.05)], and falls [21.8% vs. 25.4% non-HNI, (*p* < 0.05)] but more interpersonal violence [3.2% vs. 2.2% non-HNI, (*p* < 0.05)], bicycle use [15.3% vs. 8.2%, (*p* < 0.05)] and pedestrian use [7.8% vs. 5.4% non-HNI, (*p* < 0.05)]. An alcoholic influence was documented in 11.1% of non-HNI patients and was higher in HNI patients (17.0%), (*p* < 0.05).

The injury severity score (ISS) was 17.9 for non-HNI patients and 21.3 for HNI patients, (p < 0.05). The non-HNI patients had mortality rates of 10.6% compared with 11.5% for HNI patients (p > 0.05). The average duration of hospitalization was 15.6 days in the non-HNI group, with 5.8 days in the intensive care unit, whereas in the HNI group, the average hospital stay was 16.2 days, with 7.6 days in the ICU.

The concomitant injuries were stratified according to the AIS severity score. An injury with an AIS severity score ≥ 3 is considered serious. A total of 51.7% of the patients with head and neck injury had AIS scores ≥ 3, while 31.4% of the non-HNI patients had AIS scores ≥ 3 (*p* < 0.05)). In other regions, the same rate of severe injury was lower in the HNI group than in the non-HNI group: thorax (39.5% of patients vs. 35.4% of HNI patients), abdomen (10.8% vs. 6.7% of HNI patients, (*p* < 0.05)), and extremities (25.6% vs. 20.1%, (*p* < 0.05)). After segregation into smaller regions, the same tendency was observed for the spine (29.8% non-HNI patients vs. 27.0% HNI patients), lower extremities (26.1% vs. 22.3%, respectively), and iliac bone (16.5% non-HNI patients vs. 13.6% HNI patients), but the opposite trend was observed for the upper extremities (29.0% non-HNI patients and 33.8% HNI patients), (*p* < 0.05).

In the context of spinal trauma, the cervical spine was injured in 14.9% of HNI patients and in 12.0% of other patients, (*p* < 0.05). In the thoracic spine, 13.9% of injuries occurred in non-HNI patients compared with 12.1% of HNI patients; in the lumbar spine, 15.4% of injuries occurred in non-HNI patients and 9.9% occurred in HNI patients, (*p* < 0.05).

### Results from the dortmund maxillofacial trauma registry

The data from the Dortmund Maxillofacial Trauma Registry were collected between 2007 and 2017. A total of 7010 patients were admitted to the hospital for head and neck injuries. Among these patients, 289 patients were severely injured and presented with concomitant injuries (thorax, abdomen 4.1%). The yearly admission rate varies between 15 and 41 patients per year, and the trend was increasing. The emergency room team was alarmed for 101 patients (34.9%). A total of 236 patients (81.7%) presented with traumatic brain injury. Forty-three patients (14.7%) were transported with ventilation. As per the guidelines, 68 of the 289 patients (23.5%) were considered polytrauma patients.

In the whole patient population (7010 patients), the overall hospital stay was 2.6 ± 3.3 days; in the population with torso injuries, it was as long as 5.9 ± 12.4 days, (*p* < 0.05). This difference was statistically significant (*t* test, *p* < 0.001). A total of 199 males and 90 females were analysed (68.9% and 31.1%, respectively, (*p* < 0.05)). Therefore, the male-to-female ratio was 2.2:1. A total of 68 patients, including 55 males (80.9%) and 13 females (19.1%), suffered polytrauma. The male-to-female ratio in this group was 4.2:1. In the non-polytrauma group (221 patients), 144 males (65.2%) and 77 females (34.8%) were included, and the male-to-female ratio was 1.87:1. The average age of the study subjects was 50.5 ± 23.9 years: 47.3 ± 21.8 years for males and 57.4 ± 27 years for females. (Supplementary Table 1) No in-hospital deaths were reported in this population. Seven of the 289 patients (2.4%) needed reanimation upon arrival in the emergency room. Six of them were male, and one was female. All of them presented with traumatic brain injury. The average scores of the reanimated patients were ISS = 50.3 and GCS = 7.1. Six of the 7 patients had a calvarial fracture (85.7%), and injuries to the cervical, thoracic and lumbar spine were detected in 1, 2 and 2 patients (14.3%, 28.6% and 28.6%, respectively). Ventilation was applied in 4 patients (57.1%).

The aetiologies of the injuries included falls (49.5%, 143 cases), road traffic accidents (75, 26.0%), interpersonal violence (48, 16.6%), work-related accidents (14, 4.8%), sports accidents (7, 2.4%) and other causes (2, 0.7%). In the case of falls, the highest rate ranged from 42.3% for complex injuries to 62.5% for forehead injuries. Road traffic accidents resulted in the highest rate of dentoalveolar injuries (60%) and the lowest rate of lateral midface fractures (17.1%). Other causes were responsible for the injury in 0.0% to 30% of patients *(Supplementary Tables 2 and 3).*

**Fig. 2:**
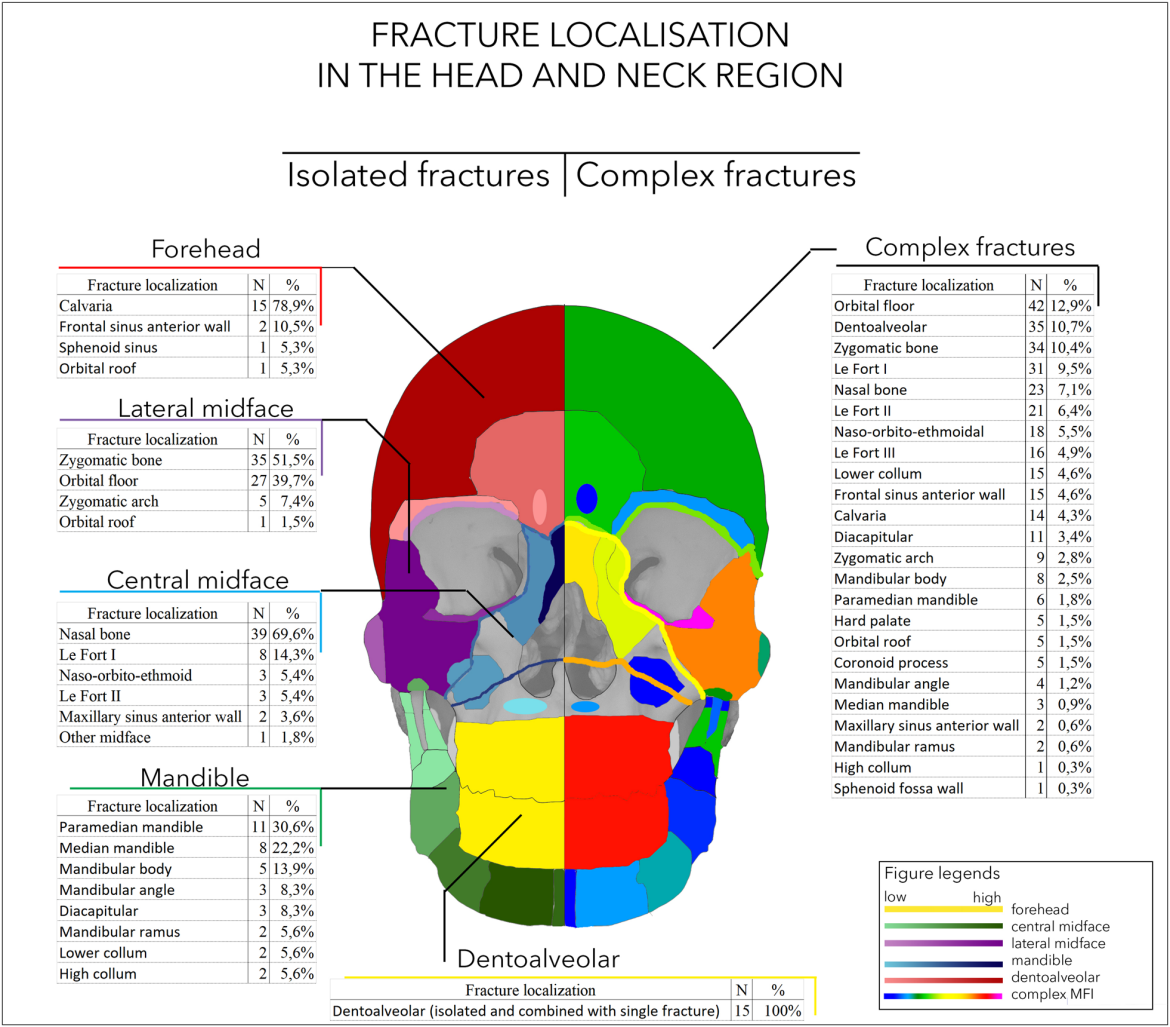
Summary of head and neck fractures in the study population. Each patient group is marked with different colours on the left side. The fracture sites in patients with complex facial fractures are presented on the right side of the figure. The grey areas mark regions without actual fracture lines (which are present in the whole head and neck trauma population)

The stratification of the patients according to head and neck injury location was performed (Fig. 2). The heatmap presents the different numbers of fractures in each region. The right side of the picture represents the distribution of fractures in various regions in patients with complex fractures.

**Fig. 3:**
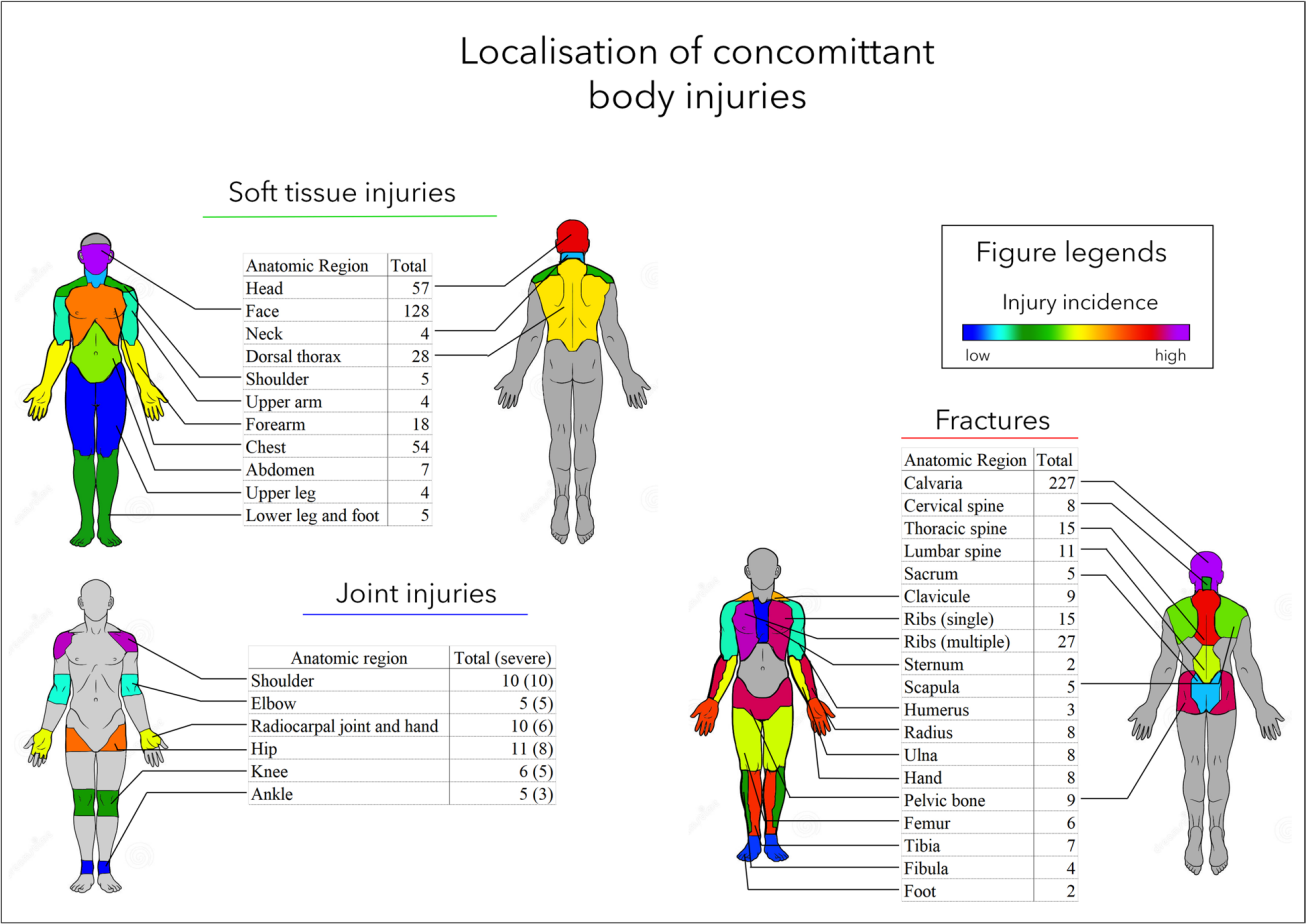
Summary of body-wide injuries as concomitant injuries to the head and neck injuries. The injuries to different body regions were grouped as “soft tissue injuries”, including the smallest superficial injuries to injuries of any size and depth; “joint injuries”, from the easiest trauma to fractures of the joint surfaces; and “fractures” of the different bony structures

The different types of body-wide injuries are summarized in Fig. 3. Most of the soft tissue injuries were identified in the face (128) and head areas (57), followed by the thorax and back areas (54 and 28 wounds, respectively). Most joint injuries were observed in the hip, shoulder radiocarpal, and hand regions (11, 10, and 10). Among these injuries, most were considered severe.

The body-wide fracture distribution was similar: most fractures occurred in the calvaria (227) and thoracic areas (27 multiple rib fractures and 15 single rib fractures). The rates of fractures in the thoracic spine (15), lumbar spine (11), and pelvic bone (9) were similarly high. In this patient group, fractures of the bones of the extremities were generally less common.

**Fig. 4:**
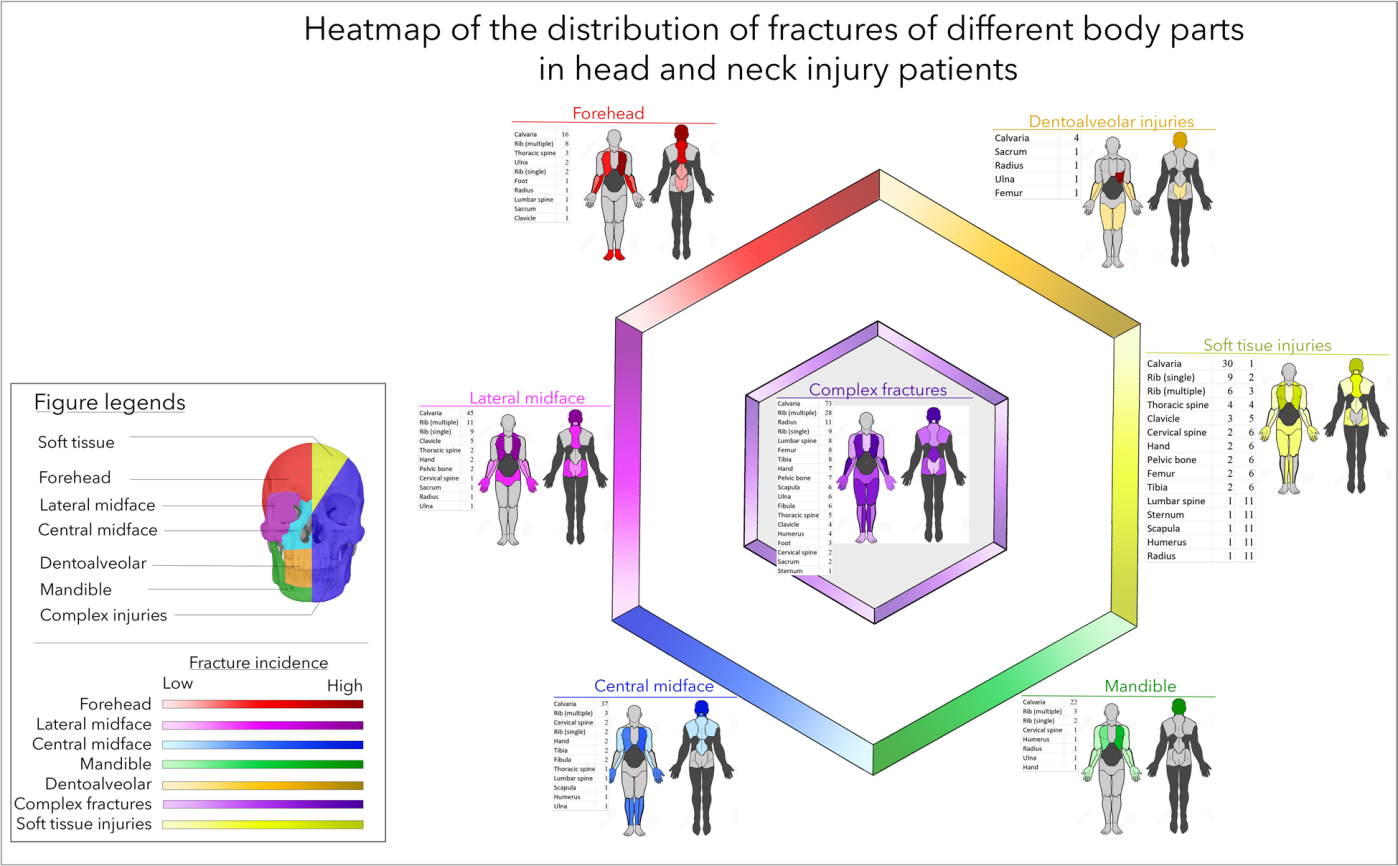
Breakdown of the associated fractures in all patient groups with head and neck injuries. Notably, in each group, concomitant injuries to the cranial area presented the highest frequency. Forehead, lateral midfacial, and mandibular injuries more frequently presented with concomitant cranial injuries, and central midface and dentoalveolar injuries more frequently presented with injuries to the lower extremities. In the case of complex fractures and patients with head and neck soft tissue injuries, the distribution was the most balanced in terms of the craniocaudal relationship; however, in both cases, important central structures were mostly endangered (thorax, lumbar spine, and femur)

Figure 4 presents the distribution of fractures in patient groups with different maxillofacial injuries. Soft tissue injuries, complex fractures, and forehead and lateral midface fractures were more likely to be associated with central injuries, which included injuries to the whole spine, ribs, and hip. Mandibular injuries were related to fractures of the lateral thoracic region and upper extremities. Central midfacial fractures were likely to co-occur with injuries to the legs, ribs, and upper extremities, which were less likely to be associated with those of the back.

**Fig. 5:**
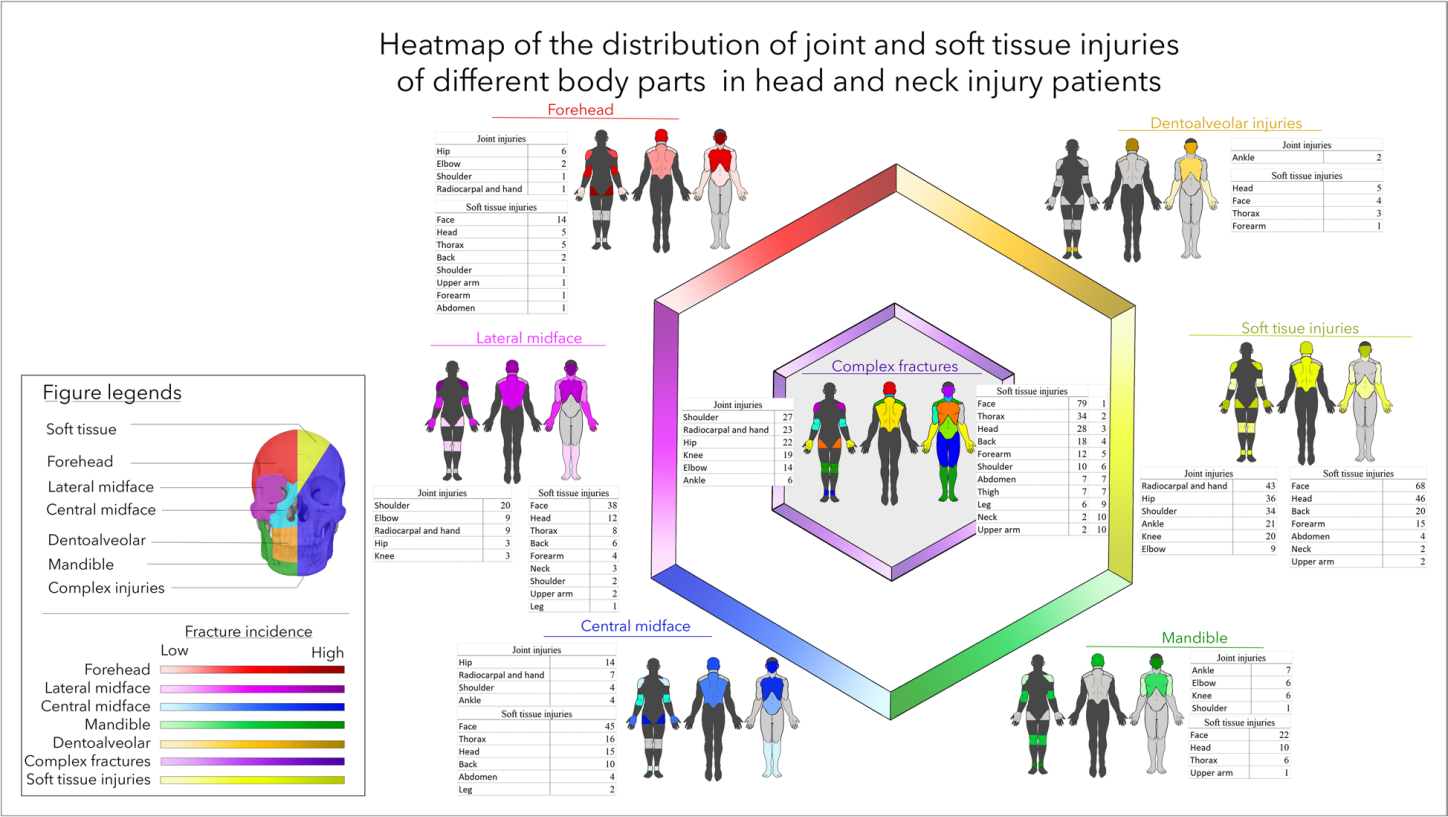
Breakdown of concomitant soft tissue and joint injuries in each head and neck injury group. The distribution of concomitant injuries is very similar to the fracture distribution shown in Fig. 4

A very similar distribution to the fracture pattern could be observed in Fig. 5 presents the localization of joint and soft tissue injuries associated with HNI in the different regions. In patients with dentoalveolar and mandibular fractures, peripheral injuries dominated. Complex fractures and lateral midface fractures were mostly associated with a central injury pattern.

## Discussion

This study has found that a significantly higher portion of patient with head and neck injury suffer severe injury and have a higher mortality rate, too. Both the data from the German Trauma Registry and our trauma registry confirm this finding. Patients with head and neck trauma were more likely to be transported to primary trauma centres. A total of 20% of all injured patients presented with HNI [24]. HNI patients had higher ISSs, needed both longer ICU and hospital stays, had higher mortality risks.

The aetiology differed from that of other patients. Fifty percent more (11.1% in the non-HMI group vs. 17% in the HNI group) patients were registered with an alcohol influence. Interpersonal violence, walking, and bicycle usage were associated with an increased risk. Suicide, motorcycle, or car accidents played a less meaningful role.

The HNI patients showed a different overall distribution of concomitant injuries: more injuries occurred in the cervical spine and upper extremities than in the general injured population. Other regions, such as the thoracic or lumbar spine, iliac bone, and lower extremities, tended to be less involved in HNI. The greater the relative distance to the head, the fewer injuries reported. These findings concur with our findings in this paper and with those of studies of cervical spine injuries (in Bicsák et al. [1]).

In our experience, well-visible head and neck injuries are often first identified by emergency personnel. In such cases, if the initial assessment is redundant, concomitant injuries might be detected later, which endangers the patients. This group of patients with torso injuries accounts for 4.1% of the total population with MFI. However, this population is only a more minor part of our trauma patients; they suffer more severe injuries and require significantly more extended hospital stays and, therefore, more attention. Similar results have also been reported in the literature [5, 15, 16, 25, 26].

This study revealed some patterns of the associated injuries. This topic is, however, less frequently covered in the literature [27]. The most common concomitant injuries were injuries to the neurocranium and traumatic brain injuries, both as soft tissue injuries and fractures. After patients were grouped based on their HNI pattern, patients with soft-tissue HNI presented relatively even fractures and soft-tissue injuries throughout the whole body. Patients with complex fractures of the head and neck presented with the most complex full-body fractures and joint and soft tissue injury patterns. In addition, these patient groups included the most subjects with polytrauma. In the case of forehead injuries, an alarming pattern of spine injuries both in the cervical spine and in more caudal sections of the spine was observed. The pattern was similar in patients with lateral midfacial fractures, but the fractures shifted caudally, and the pelvis became involved. However, no pelvic fractures were detected, yet in many cases, femoral fractures occurred. Isolated dentoalveolar fractures were associated with central neurotrauma and thorax injuries. Otherwise, we observed only injuries to the extremities. The joint injuries and soft tissue injury locations were very similar.

As the above pattern shows, patients with complex HNIs and lateral midfacial fractures were most likely to have central injuries; however, caution is suggested in treating patients with only soft tissue HNIs. Importantly, in this study, we found 15 injuries to the thoracic spine and 11 injuries to the lumbar spine. Moreover, we reported 173 patients with CSI [20], with an approximately 6.7-fold higher incidence in this latter patient group.

The studies of Yang et al. [19] showed in animal experiments that not only the trauma severity (described by the ISS) but also the trauma pattern were fundamental and determine the outcome. Similar clinical observations have been reported by many groups [5, 25, 28, 29]. The above heatmaps revealed coherence in HNI and torso injury patterns. The numbers of patients are smaller than those of patients with CSI [20]. Therefore, statistical significance can be less well-analysed. Nevertheless, the tendency is clearly presented for all injury types, including soft tissue injuries, joint injuries, and fractures.

The 289 analysed patients accounted for 4.1% of all patients with head and neck injuries. Together with the 2.47% of patients with CSI, all HNI patients with any central body injury accounted for nearly 6.5% of all HNI patients. This number is not very high. However, many of these injuries are potentially life-threatening. Emergency service personnel, emergency room personnel and all cooperating hospital departments should be aware of this fact. A full-body check upon the patient’s arrival must be performed in all ER protocols to avoid missing diagnoses.

A limitation of the study is that a part of the study patients, who are eligible for the report to the Trauma Register DGU® are also considered in this sample. As this is far below the statistical margin of error, we decided not to consider this in the statistical process.

## Conclusion

Patients with head and neck injuries tend to have slightly different injury pattern in the whole body than patients without head and neck injuries. Head and neck injuries in different locations show different patterns of concomitant injuries. Considering the most alarming injuries, panfacial fractures, forehead and lateral and central midfacial injuries and spine injuries tend to combine with each other. Central midface, mandibular and dentoalveolar injuries were not observed with hip fracture together. Injuries to the thorax are common in nearly all HNI cases but were not diagnosed in case of dentoalveolar injuries.

Detecting injury patterns can lead to improved diagnostics and can improve patient safety.

Our database is comparable with the databank of the Trauma Register DGU® and, therefore, can be considered that it represents a random sample for a much bigger population.

## Supplementary Information

Supplementary material 1

## Data Availability

Availability of data The data that support the findings of this study are available from the corresponding author upon reasonable request.

## Abbreviations

AIS: Abbreviated injury score
AO: Allgemeine Osteosynthesegruppe (AO Foundation, Davos, CH)
AUC: Akademie der Unfallchirurgie, Training organisation oft he DGU
CSI: Cervical spine injury
DGU: Deutsche Gesellschaft für Unfallchirurgie (German Trauma Society)
GCS: Glasgow coma scale
HNI: Head and neck injury
ISS: Injury severity score
MFI: Maxillofacial injury
SD: Standard deviation
