# Supplementary material for: Concomitant body-wide trauma patterns in patients with head and neck injuries: a comparison based on the trauma register DGU® by the German trauma society and the dortmund maxillofacial trauma registry
Source: Eur J Med Res. 2025 May 8;30:371. doi: 10.1186/s40001-025-02636-x (PMC12060487; doi:10.1186/s40001-025-02636-x)
Supplement: Supplementary file 1 — Supplementary material 1 [file 40001_2025_2636_MOESM1_ESM.docx]

Supplementary data

Supplementary tables 1-5

Supplementary Table 1 Presents the age and gender distribution among patients with different levels of HNI. All age data and SD data are presented as years, N as absolute numbers. Statistically significant p-values are marked in Italics.

| MFI level | Male | | | Female | | | Total | | | p-value |
| --- | --- | --- | --- | --- | --- | --- | --- | --- | --- | --- |
|  | **Average age** | **N** | **SD** | **Average age** | **N** | **SD** | **Average age** | **N** | **SD** |  |
| Soft tissue | 53.3 | 45 | 25.7 | 59.7 | 38 | 27.1 | 56.2 | 83 | 26.4 | p=0.277 |
| Forehead | 34.6 | 11 | 26.3 | 47.1 | 5 | 34.8 | 38.5 | 16 | 28.6 | *p=0.034* |
| Central midface | 51.8 | 26 | 21.9 | 14 | 55.8 | 28.9 | 40.0 | 53.2 | 24.3 | *p=0.007* |
| Lateral midface | 45.2 | 14 | 55.8 | 65.6 | 19.2 | 21.0 | 54.3 | 20.4 | 47 | p=0.058 |
| Midface all | 48.5 | 52 | 19.5 | 61.7 | 35 | 23.7 | 53.8 | 87 | 22.1 | *p=0.010* |
| Mandible | 43.6 | 18 | 16.9 | 31.2 | 4 | 20.4 | 41.3 | 22 | 17.7 | *p<0.005* |
| Complex fractures | 45.5 | 70 | 19.9 | 53.4 | 6 | 35.3 | 46.1 | 76 | 21.3 | *p<0.005* |
| Dentoalveolar | 50.4 | 3 | 31.7 | 27.25 | 2 | 14.2 | 41.14 | 5 | 26.7 | p=0.527 |

Supplementary Table 2: Summary of etiology factors per HNI level and percentage of injuries at each HNI level. Absolute numbers and percentages (%) are provided.

|  | **Soft tissue** | | **Fore-head** | | **Central midface** | | **Lateral midface** | | ***Midface (all)*** | | **Mandible** | | **Complex** | | **Dentoalveolar** | |
| --- | --- | --- | --- | --- | --- | --- | --- | --- | --- | --- | --- | --- | --- | --- | --- | --- |
| **Road traffic accident** | 21 | 25.3% | 5 | 31.3% | 10 | 25.0% | 8 | 17.0% | *18* | 20.7% | 5 | 22.7% | 24 | 31.6% | 2 | 40.0% |
| **Fall** | 45 | 54.2% | 10 | 62.5% | 18 | 45.0% | 24 | 51.1% | *42* | 48.3% | 11 | 50.0% | 32 | 42.1% | 3 | 60.0% |
| **Sports accident** | 3 | 3.6% | 0 | 0.0% | 0 | 0.0% | 2 | 4.3% | *2* | 2.3% | 2 | 9.1% | 0 | 0.0% | 0 | 0.0% |
| **Work related accident** | 1 | 1.2% | 1 | 6.3% | 0 | 0.0% | 2 | 4.3% | *2* | 2.3% | 0 | 0.0% | 10 | 13.2% | 0 | 0.0% |
| **Interpersonal violence** | 13 | 15.7% | 0 | 0.0% | 12 | 30.0% | 11 | 23.4% | *23* | 26.4% | 3 | 13.6% | 9 | 11.8% | 0 | 0.0% |
| **Other/unknown** | 0 | 0.0% | 0 | 0.0% | 0 | 0.0% | 0 | 0.0% | *0* | 0.0% | 1 | 4.5% | 1 | 1.3% | 0 | 0.0% |
| **Total** | ***83*** | 100.0% | ***16*** | 100.0% | ***40*** | 100.0% | ***47*** | 100.0% | ***87*** | 100.0% | ***22*** | 100.0% | ***76*** | 100.0% | ***5*** | 100.0% |

Supplementary table 3. Presentation of the highest percentage of etiology in different maxillofacial injury levels.

| **Etilogy** | **Maxillofacial region** | **%** |
| --- | --- | --- |
| **Road traffic accident** | Dentoalveolar | 40.0% |
| **Fall** | Forehead | 62.5% |
| **Sports accident** | Mandible | 9.1% |
| **Work related accident** | Complex fractures | 13.2% |
| **Interpersonal violence** | Central midface | 30.0% |

Supplementary table 4. Percentage data (Injury Rate, IR=%) to Figure 3. Column A (left) represents the bodywide fracture sites, Column B (middle) represents the joint injuries, and Column C (right) the soft tissue injuries.

| **Fractures** | **IR** |  | **Joint injuries** | **IR** |  | **Soft tissue injuries** | **IR** |
| --- | --- | --- | --- | --- | --- | --- | --- |
| Sacrum | 3.8% |  | Shoulder right | 14.5% |  | Head right | 6.9% |
| Clavicle left | 1.7% |  | Shoulder left | 15.6% |  | Head left | 19.7% |
| Clavicle right | 3.1% |  | Elbow right | 7.3% |  | Face right | 22.1% |
| Rib left | 1.4% |  | Elbow left | 7.3% |  | Face left | 44.3% |
| Rib right | 5.2% |  | Hand left | 6.6% |  | Neck right | 49.1% |
| Ribs (series) left | 6.2% |  | Hand right | 15.2% |  | Neck left | 1.4% |
| Ribs (series) right | 9.3% |  | Hip right | 13.5% |  | Back right | 1.0% |
| Sternum | 11.1% |  | Hip left | 15.6% |  | Back left | 9.7% |
| Scapula left | 0.7% |  | Knee right | 12.5% |  | Shoulder right | 9.7% |
| Scapula right | 1.7% |  | Knee left | 7.3% |  | Shoulder left | 1.7% |
| Humerus left | 1.0% |  | Ankle right | 9.3% |  | Upper arm right | 1.7% |
| Humerus right | 1.0% |  | Ankle left | 6.9% |  | Upper arm left | 1.4% |
| Radius left | 1.4% |  |  |  |  | Lower arm right | 1.4% |
| Radius right | 2.8% |  |  |  |  | Lower arm left | 6.2% |
| Ulna left | 2.8% |  |  |  |  | Chest right | 5.2% |
| Ulna right | 2.8% |  |  |  |  | Chest left | 18.7% |
| Hand left | 1.4% |  |  |  |  | Stomach right | 18.0% |
| Hand right | 2.8% |  |  |  |  | Stomach left | 2.4% |
| Pelvis left | 2.1% |  |  |  |  | Thigh right | 3.1% |
| Pelvis right | 3.1% |  |  |  |  | Thigh left | 1.4% |
| Femur left | 3.8% |  |  |  |  | Leg, foot right | 1.0% |
| Femur right | 2.1% |  |  |  |  | Leg, foot left | 1.7% |
| Tibia left | 1.7% |  |  |  |  |  |  |
| Tibia right | 2.4% |  |  |  |  |  |  |
| Fibula left | 2.4% |  |  |  |  |  |  |
| Fibula right | 1.4% |  |  |  |  |  |  |
| Foot left | 1.4% |  |  |  |  |  |  |
| Foot right | 0.7% |  |  |  |  |  |  |

Supplementary table 5. Distribution of bodywide fractures on the left and right side. P-value represents the comparison of the fractures on both sides with χ^2^-test (significance: p<0.05). Grey indicates the cells/body regions in which a comparison is not applicable.

| **Anatomic region** | **Left** | **Right** | **Total** | **Rank total** | **p-value** | **% of all fractures** |
| --- | --- | --- | --- | --- | --- | --- |
| Clavicle | 9 | 4 | 13 | 9 | 0.050 | 4.5% |
| Rib (single) | 15 | 18 | 33 | 3 | 0.460 | 11.4% |
| Ribs (serial) | 27 | 32 | 59 | 2 | 0.357 | 20.4% |
| Scapula | 5 | 3 | 8 | 13 | 0.317 | 2.8% |
| Humerus | 3 | 4 | 7 | 16 | 0.593 | 2.4% |
| Radius | 8 | 8 | 16 | 5 | 1.000 | 5.5% |
| Ulna | 8 | 4 | 12 | 10 | 0.103 | 4.2% |
| Hand | 8 | 6 | 14 | 7 | 0.450 | 4.8% |
| Pelvic bone | 9 | 11 | 20 | 4 | 0.527 | 6.9% |
| Femur | 6 | 5 | 11 | 11 | 0.670 | 3.8% |
| Tibia | 7 | 7 | 14 | 7 | 1.000 | 4.8% |
| Fibula | 4 | 4 | 8 | 13 | 1.000 | 2.8% |
| Foot | 2 | 2 | 4 | 18 | 1.000 | 1.4% |
| Calvaria |  |  | 227 | 1 |  | 78.5% |
| Cervical spine |  |  | 8 | 13 |  | 2.8% |
| Thoracal spine |  |  | 15 | 6 |  | 5.2% |
| Lumbar spine |  |  | 11 | 11 |  | 3.8% |
| Sacrum |  |  | 5 | 17 |  | 1.7% |
| Sternum |  |  | 2 | 19 |  | 0.7% |
